# Effect of Calomelanone, a Dihydrochalcone Analogue, on Human Cancer Apoptosis/Regulated Cell Death in an *In Vitro* Model

**DOI:** 10.1155/2020/4926821

**Published:** 2020-12-19

**Authors:** Wasitta Rachakhom, Ratana Banjerdpongchai

**Affiliations:** Department of Biochemistry, Faculty of Medicine, Chiang Mai University, Chiang Mai 50200, Thailand

## Abstract

Calomelanone, 2′,6′-dihydroxy-4,4′-dimethoxydihydrochalcone, possesses anticancer activities. This study was conducted to investigate the cytotoxic effect of calomelanone, a dihydrochalcone analogue, on human cancer cells and its associated mechanisms. The cytotoxic effect of calomelanone was measured by MTT assay. Annexin V-FITC/propidium iodide and DiOC_6_ staining that employed flow cytometry were used to determine the mode of cell death and reduction of mitochondrial transmembrane potential (MTP), respectively. Caspase activities were measured using specific substrates and colorimetric analysis. The expression levels of Bcl-2 family proteins were determined by immunoblotting. Reactive oxygen species were also measured using 2′,7′-dihydrodichlorofluorescein diacetate and dihydroethidium (fluorescence dyes). Calomelanone was found to be toxic towards various human cancer cells, including acute promyelocytic HL-60 and monocytic leukemic U937 cells, in a dose-dependent manner at 24 h and human hepatocellular HepG2 cells at 48 h. However, the proliferation of HepG2 cells increased at 24 h. Calomelanone was found to induce apoptosis in HL-60 and U937 at 24 h and HepG2 apoptosis at 48 h via the intrinsic pathway by inducing MTP disruption. This compound also induced caspase-3, caspase-8, and caspase-9 activities. Calomelanone upregulated proapoptotic Bax and Bak and downregulated antiapoptotic Bcl-xL proteins in HepG2 cells. Moreover, signaling was also associated with oxidative stress in HepG2 cells. Calomelanone induced autophagy at 24 h of treatment, which was evidenced by staining with monodansylcadaverine (MDC) to represent autophagic flux. This was associated with a decrease of Akt (survival pathway) and an upregulation of Atg5 (the marker of autophagy). Thus, calomelanone induced apoptosis/regulated cell death in HL-60, U937, and HepG2 cells. However, it also induced autophagy in HepG2 depending on duration, dose, and type of cells. Thus, calomelanone could be used as a potential anticancer agent for cancer treatment. Nevertheless, acute and chronic toxicity should be further investigated in animals before conducting investigations in human patients.

## 1. Introduction

Among the primary forms of liver cancer, hepatocellular carcinoma (HCC) is the most commonly diagnosed primary malignant tumor of the liver with high rates of incidence around the world [1]. The risk factors for primary liver cancer include hepatitis B virus and hepatitis C viral infection, alcohol consumption, tobacco, oral contraceptives, and aflatoxin [2]. Leukemia is a cancer of the hematopoietic system resulting in abnormal proliferation of white or red blood cells [3]. Leukemia is frequently found in childhood and is known to be caused by genetic, radioactive, infectious, and environmental factors [4]. Cancer can be treated with surgery, radiation, chemotherapy, and immune therapy. For the treatment of leukemia, patients need to receive chemotherapy, radiation, or bone marrow transplants. The goal of these treatments is to induce cancer cell death through regulated cell death.

The three major types of regulated cell death are composed of apoptosis, autophagic cell death, and necroptosis [5]. The suppression of apoptosis during tumorigenesis plays an important role in the development and progression of cancer [6]. There are two main pathways of apoptosis, namely, the mitochondria-mediated or intrinsic pathway and the death receptor or extrinsic pathway [7]. Notably, caspases also play an important role in apoptotic cell death signaling [8].

Autophagy differs from apoptosis, and it is activated by various conditions enabling it to maintain nutrient levels during amino acid deprivation for cell survival [9]. The process of autophagy consists of a number of steps. The phagophore is formed by activating Beclin-1 and PI3K class III, whereas Atg12, Atg5, and LC3 serve as a complex control in the formation of the autophagosome. The fusion of the autophagosome with the lysosome is followed by proteolytic degradation (hydrolase) at an acidic pH [10]. Recently, it has been reported that the PI3K/Akt/mTOR pathway [11] is involved in the autophagy signaling pathway. Autophagy is further regulated by mTOR signaling, which can be inhibited by several forms of stress, including hypoxia, oxidative stress, pathogen infection, and nutrient starvation conditions [12], and by an autophagy inducer such as rapamycin [13].

In accordance with the evolution of cancer resistance and the adverse side effects of chemotherapeutic drugs and targeted therapies, a novel strategy for cancer treatment is needed. A recent study has reported that chalcone and dihydrochalcone display a high potential to be used in cancer treatments [14]. Dihydrochalcones belong to a group of bicyclic flavonoids that are identified by the presence of a benzylacetophenone. Dihydrochalcones consist of two benzene rings joined with a saturated three-carbon bridge [15]. Dihydrochalcones and their derivatives possess many biological activities such as anti-inflammatory, antioxidant, antimicrobial, and especially anticancer activities [16]. A member of the dihydrochalcone group, cryptocaryone, induces apoptosis via the death receptor in human prostate cancer cells [17]. Furthermore, 3,4′-trimethoxy-2′-hydroxy-chalcone (CH1) and 3′-bromo-3,4-dimethoxy-chalcone (CH2), which are chalcone derivatives, induce HepG2 cell apoptosis via the intrinsic pathway by inducing caspase-3 and caspase-9 activities [18]. Phloretin, 2′,4,4′,6′-tetrahydroxydihydrochalcone, induces human promyelocytic leukemic HL-60 cell apoptosis via inhibition of protein kinase C activity [19].

**Figure 1:**
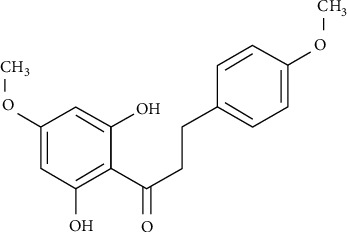
Chemical structure of calomelanone used in this study (2′,6′-dihydroxy-4,4′-dimethoxydihydrochalcone).

The dihydrochalcone analogues, calomelanone (2′,6′-dihydroxy-4,4′-dimethoxy-dihydrochalcone, DHC-1) (Figure 1) and 4′,6′-dihydroxy-2′,4-dimethoxy-5′-(2^″^-hydroxybenzyl)-dihydrochalcone (DHC-2) (Supplemental Figure 1(a)), have been classified within the group of dihydrochalcones. DHC-2 was reported to induce apoptosis in breast cancer cells, particularly MDA-MB-231 and MCF-7 [20]. However, with regard to calomelanone, a derivative of dihydrochalcone, there have been no published studies on its cytotoxicity, mode of cell death, and its relevant mechanism(s) in human liver cancer and leukemic cell models. Thus, the aims of this study were to investigate the cytotoxic effects of calomelanone on human hepatocellular carcinoma HepG2, acute promyelocytic leukemic HL-60, and monocytic leukemic U937 cells along with the associated mechanism(s). It was found that calomelanone could induce apoptosis via both the intrinsic and extrinsic pathways, as well as through the activities of caspases in all three cell lines. The compound was also found to promote HepG2 cell apoptosis through oxidative stress and the alteration of Bcl-2 family protein levels. Moreover, the compound was also found to be able to induce autophagy at 24 h via the upregulation of Atg5 and the downregulation of Akt proteins.

## 2. Materials and Methods

### 2.1. Chemicals

Calomelanone, 2′,6′-dihydroxy-4,4′-dimethoxydihydrochalcone (Figure 1) (product code: FD65868), was purchased from Carbosynth Ltd. (Berkshire, UK). Dulbecco's Modified Eagle's Medium (DMEM), fetal bovine serum (FBS), streptomycin, and penicillin G sodium were obtained from Gibco BRL (Thermo Fisher Scientific Inc., Waltham, MA, USA). Dimethyl sulfoxide (DMSO), 3-(4,5-dimethyl)-2,5-diphenyl tetrazolium bromide (MTT), 4-(2-hydroxyethyl)-1-piperazineethanesulfonic acid (HEPES), propidium iodide (PI), 2′,7′-dihydrodichlorofluorescein diacetate (DCFH-DA), dihydroethidium (DHE), Ficoll-Hypaque-1077, and 3,3′-dihexyloxacarbocyanine iodide (DiOC_6_) were obtained from Sigma-Aldrich (St. Louis, MO, USA). The Annexin V-FITC-FLUOS kit (REF11988549001) and protease inhibitor cocktail tablets (11697498001) were obtained from Merck (Darmstadt, Germany). Caspase Colorimetric Assay Kits including the substrates of caspase-9, LEHD-*para*-nitroaniline (LEHD-*p*-NA, Cat: 68CL-Casp9-S400), caspase-8, IETD-*para*-nitroaniline (IETD-*p*-NA, Cat: 68CL-Casp8-S400), and caspase-3, DEVD-*para*-nitroaniline (DEVD-*p*-NA, Cat: 68CL-Casp3-S200) were obtained from RayBiotech, Inc. (Peachtree Corners, GA, USA). The Autophagy Assay Kit (ab139484); the primary antibodies against Bax (ab53154), Bak (ab32371), Bcl-xL (ab98143), Atg5 (ab109490), Akt (ab32505), and *β*-actin (ab8227); and the secondary antibodies for goat anti-rabbit IgG-horseradish peroxidase (HRP) (ab97051) and goat anti-mouse IgG-HRP (ab97046) were purchased from Abcam (Cambridge, UK).

### 2.2. Cell Culture

The human hepatocellular carcinoma (HepG2) cell line was obtained from the Japanese Collection of Research Bioresources (JCRB) Cell Bank, Japan. Human promyelocytic leukemic HL-60 cells and human monocytic leukemic U937 cells were gifts from Professor Sukathida Ubol (Department of Microbiology, Faculty of Science, Mahidol University) and Professor Watchara Kasinroek (Faculty of Associated Medical Science, Chiang Mai University), respectively. Cells were cultured in DMEM, supplemented with 10% FBS, 25 mM (NaHCO_3_), 20 mM HEPES, 100 units/ml penicillin G, and 100 *μ*g/ml streptomycin, and then maintained at 37°C with 5% CO_2_ in a humidified atmosphere [21].

Peripheral blood mononuclear cells (PBMCs) were isolated from the buffy coated part of whole blood obtained from the Blood Donation Center, Maharaj Nakorn Chiang Mai Hospital, Chiang Mai University, Chiang Mai, Thailand. All donors provided their informed consent. The cells in the buffy coat were separated by Ficoll-Hypaque following density gradient centrifugation. Briefly, the whole blood was diluted with 1 × volume of phosphate-buffered saline (PBS, pH 7.2). Twenty milliliters of diluted blood cells was carefully overlayered over 10 ml of Ficoll-Hypaque and then centrifuged at 400 × g for 30 min at room temperature. The upper layer was aspirated leaving the whitish mononuclear cell layer undisturbed at the interphase, and the mononuclear cell layer was then carefully removed to a new tube. PBMCs were washed twice with PBS and centrifuged at 300 × g for 10 min at room temperature. The precipitated PBMCs, HL-60, and U937 were cultured in the RPMI-1640 medium supplemented with 10% fetal bovine serum (FBS), 2 mM glutamine, 100 units/ml penicillin G, and 100 *μ*g/ml streptomycin at 37°C in a 5% CO_2_ atmosphere [20].

### 2.3. Cytotoxicity Assay

Calomelanone was solubilized in DMSO (50 mM) for stock solution and diluted with complete media to final concentrations (IC_10_, IC_20_, and IC_50_) for treatment in all experiments. HepG2 cells (5 × 10^4^ cells/ml) were treated with calomelanone at various concentrations for 24, 48, and 72 h, whereas HL-60 and U937 cells (5 × 10^4^ cells/ml) were incubated for 24 h. Peripheral blood mononuclear cells (PBMCs, 3 × 10^6^ cells/ml) were treated with calomelanone for 24 and 48 h. Percent cell viability was measured using MTT assay, and the results were compared with those of the untreated cells. Briefly, after incubation, MTT dye was added to the cell suspension at a final concentration of 100 *μ*g/ml. After incubation for 4 h at 37°C with 5% CO_2_ in a humidified atmosphere, the medium was then discarded and soluble MTT dye is changed to be the purple formazan crystals by mitochondrial succinate dehydrogenase in living cells, which then were dissolved in DMSO. The absorbance was measured at 540 nm, and the reference wavelength was recorded at 630 nm using a microplate reader (BioTek, Winooski, VT, USA). Percent cell viability was determined according to the formula described below. In this study, inhibitory concentrations or various IC (IC_10_, IC_20_, and IC_50_) values (equal to 90%, 80%, and 50% cell viability, respectively) were calculated by GraphPad Prism 6.0 software (purchased from GraphPad Software, Inc. (San Diego, CA, USA)) [22, 23] in order to compare the sensitivity of various cancer cells and normal PBMCs against the compound. These concentrations (IC values) were then used for further experiments.

(1) $$\begin{array}{c} \%\text{cell viability}=\frac{\text{Experiment O}{\text{D}}_{540}-{\text{OD}}_{630}}{\text{Control O}{\text{D}}_{540}-{\text{OD}}_{630}}\times 100. \end{array}$$

### 2.4. Apoptosis Assay

After 48 h of treatment, HepG2 cells were trypsinized, washed with PBS, and then stained with Annexin V-fluorescein isothiocyanate (FITC) and propidium iodide (PI) for 15 min. For HL-60 and U937 cells, the 24 h-treated cells were collected, centrifuged, washed with PBS, and then stained with Annexin V-FITC/PI for 15 min. The mode of cell death was demonstrated using a flow cytometer, and the results were then analyzed with CellQuest (software program) (Becton Dickinson, Franklin Lakes, NJ, USA) [21].

### 2.5. Determination of Mitochondrial Transmembrane Potential Disruption

The HL-60 and U937 cell lines were treated with various ICs and harvested after 24 h of calomelanone treatment, whereas HepG2 cells were treated with various ICs at 48 h and incubated for 48 h. The cells were then incubated with 3,3′-dihexyloxacarbocyanine iodide (DiOC_6_) at 40 nM (final concentration) for 15 min at 37°C. The loss of mitochondrial transmembrane potential (MTP) was measured using a flow cytometer (Becton Dickinson, Franklin Lakes, NJ, USA) [24].

### 2.6. Assay of Caspase-3, Caspase-8, and Caspase-9 Activities

Cancer cells were incubated with calomelanone at various concentrations for 24 h (HL-60 and U937 cells) and 48 h (HepG2 cells). The cells were harvested and then lysed with lysis buffer on ice for 10 min. After that, the chromogenic substrate of each caspase was added, and the cells were incubated for 2 h. The levels of caspase activity were determined using a spectrophotometric microplate reader at 405 nm (BioTek, Winooski, VT, USA) [25].

### 2.7. Western Blotting

The expression levels of proapoptotic proteins (Bax and Bak), antiapoptotic proteins (Bcl-xL), Akt (PI3K/Akt/mTOR pathway), and autophagic proteins (Atg5) were examined using specific antibodies. Briefly, the treated cells were lysed, and protein concentrations were measured using the Bradford assay kit. The proteins (15-35 *μ*g) were separated with 12% (*w*/*w*) SDS-PAGE and transferred to the nitrocellulose membrane. The nonspecific proteins were blocked with 5% nonfat milk in TBS containing 0.3% Tween-20.The membrane was then incubated with a primary antibody against each apoptotic protein consisting of Bax (1 : 1,000), Bak (1 : 5,000) and Bcl-xL (1 : 1,000); autophagic protein, Atg5 (1 : 1,000); and the survival pathway protein, Akt (1 : 1,000), and the constitutive protein, *β*-actin (1 : 20,000), according to its dilution factor. Incubation was conducted overnight at 4°C followed by supplementation of the appropriate horseradish peroxidase- (HRP-) conjugated secondary antibodies (1 : 8,000) for 2 h at room temperature. Antibodies were diluted with 5% skim milk in TTBS (Tween-20/TBS (Tris-buffered saline) pH 7.5). Specific protein bands were detected on X-ray film with the SuperSignal West Pico Chemiluminescent Substrate (Thermo Fisher Scientific Inc., Waltham, MA, USA). The intensity of the protein bands was determined using a densitometer (ImageJ, National Institutes of Health (NIH), USA) and normalized to that of the control protein *β*-actin. The values were then compared to the band of the untreated cells [26].

### 2.8. Measurement of Reactive Oxygen Species (ROS)

HepG2 cells were treated with calomelanone at various concentrations and incubated for 2 to 48 h. Hydrogen peroxide (peroxide radical) was determined by incubating cell suspension with 2′,7′-dihydrodichlorofluorescein diacetate (DCFH-DA) at a final concentration of 20 *μ*M for 30 min. Fluorescence intensity of DCF was detected using a fluorescence microplate reader at wavelengths of 485 nm (excitation) and 530 nm (emission), respectively. On the other hand, the superoxide anion radical level was measured using dihydroethidium (DHE) fluorescence dye. Cell suspension was incubated with DHE at a final concentration of 1 mM for 30 min. The cells were then washed twice with PBS, and the fluorescence intensity of E^+^ was determined using a fluorescence microplate reader (Synergy H4 Hybrid Reader, BioTek®, VT, USA) at wavelengths of 535 nm (excitation) and 635 nm (emission), respectively [27].

### 2.9. Examination of Autophagy

HepG2 cells were treated with calomelanone for 24 h at IC_10_, IC_20_, and IC_50_ (values recorded at 48 h of incubation) and then stained with monodansylcadaverine (MDC) to detect the autophagic vacuoles using the autophagy detection kit protocol. Briefly, calomelanone-treated cells were washed twice with 1x assay buffer. Subsequently, 100 *μ*l of MDC was added to the monolayer cells. After being incubated for 30 min in the dark at 37°C, the cells were gently washed with 100 *μ*l of 1x assay buffer. The morphology of the autophagic vacuoles was detected under fluorescence microscopy (ImageXpress® Micro 4 High-Content Imaging System, USA). The percentage of the autophagic vacuoles was also quantitated by MDC staining and by using a flow cytometer (Becton Dickinson, Franklin Lakes, NJ, USA).

### 2.10. Statistical Analysis

All data are expressed as mean ± standard deviation of the mean values (SD) obtained from triplicate runs of three independent experiments. All tests were administered using commercially available software, namely, GraphPad Prism 6.0 software (GraphPad Software, Inc., San Diego, CA, USA). The data were assessed with one-way analysis of variance (ANOVA) using post hoc Tukey's test. Comparisons between the two groups were assessed using Student's *t*-test. Data were considered significant when *p* value < 0.05.

## 3. Results

### 3.1. Cytotoxic Effect of Calomelanone against Various Cancer Cells and Normal PBMCs

**Figure 2:**
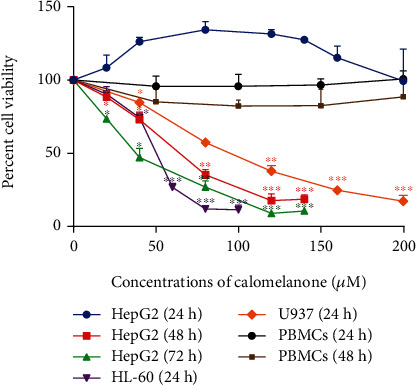
Cytotoxicity of calomelanone against various cancer cells and PBMCs. The cells were incubated with calomelanone at various concentrations for 24, 48, and 72 h. Percent cell viability was measured by MTT assay and presented as mean ± SD from triplicate runs of three independent experiments. ^∗^*p* < 0.05, ^∗∗^*p* < 0.01, and ^∗∗∗^*p* < 0.001 when compared with the control.

Since several natural compounds or derivatives are known to be toxic to cancer cells [28], the cytotoxic effect of calomelanone was determined on various cancer cells. The results indicated that calomelanone was able to inhibit HL-60 and U937 cell viability in a dose-dependent manner after incubation for 24 h, but not HepG2 cells at 24 h (Figure 2). Percent cell viability of HepG2 was decreased after calomelanone treatment at 48 and 72 h, whereas HepG2 cell viability at 24 h increased. Calomelanone was not toxic to normal PBMCs (Figure 2) indicating that it was specific to HepG2 and leukemic cells.

The inhibitory concentration values (ICs) at 10, 20, and 50% (IC_10_, IC_20_, and IC_50_) and the selectivity index (SI) of calomelanone towards various types of cells were determined and are shown in Table 1. The most sensitive cancer cell type to calomelanone treatment at 24 h (using SI) was HL-60>U937>HepG2; however, according to the IC_50_ values, the most sensitive cell type was also observed in the same sequence, viz., HL-60>U937>HepG2. The viability of calomelanone-treated HepG2 cells began to significantly decrease at 48 and 72 h. Therefore, the IC_10_, IC_20_, and IC_50_ values at 48 h were selected for further experiments. The survival of HL-60 and U937 cells significantly reduced after 24 h treatment. Thus, the IC values at this time were selected for further experiments as well.

### 3.2. Calomelanone-Induced Apoptosis in HepG2, HL-60, and U937 Cells

**Figure 3:**
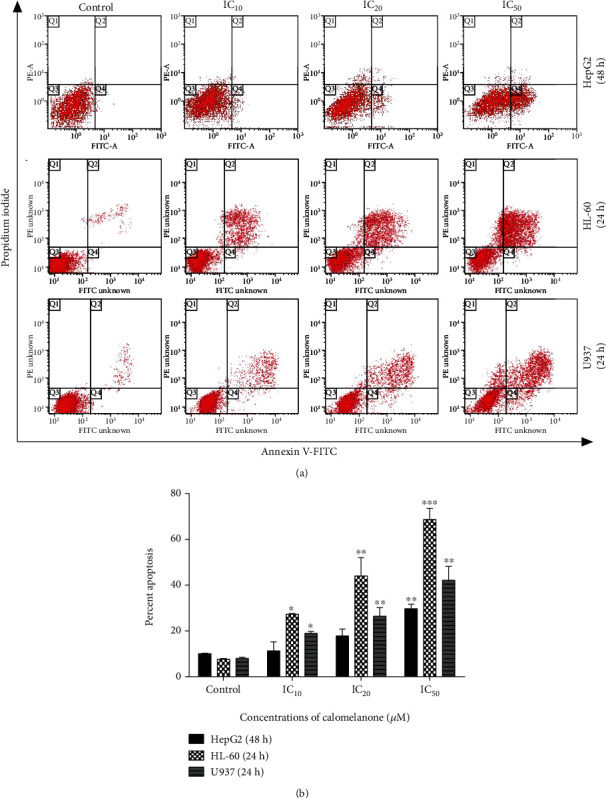
Induction of apoptosis in calomelanone-treated cancer cells. The cells were treated with the compound for 24 h (HL-60 and U937) or 48 h (HepG2) at various ICs. After treatment, cells were stained with Annexin V-FITC and propidium iodide (PI). The mode of cell death was measured using a flow cytometer. Representative dot plots of cancer cells (a) and bar graphs of percent cell apoptosis (b) are shown as mean values ± SD after the calomelanone treatment in three independent experiments. Statistical significance values compared to the control (without treatment) were marked with asterisks: ^∗^*p* < 0.05, ^∗∗^*p* < 0.01, and ^∗∗∗^*p* < 0.001 when compared with the control.

It was reported that a natural dihydrochalcone, 3-(4-hydroxyphenyl)-1-(2,4,6-trihydroxyphenyl)propan-1-one (phloretin), could induce apoptotic cell death in various types of cancer [29]. Subsequently, the mode of cell death induced by calomelanone in the three cancer cell lines, i.e., HepG2, HL-60, and U937 cells, was investigated and it was determined that the compound significantly induced the apoptotic cell death population (right lower (Q2) and right upper (Q4) quadrants) in a dose-dependent manner (Figures 3(a) and 3(b)).

### 3.3. Reduction of Mitochondrial Transmembrane Potential (MTP) Induced by Calomelanone

**Figure 4:**
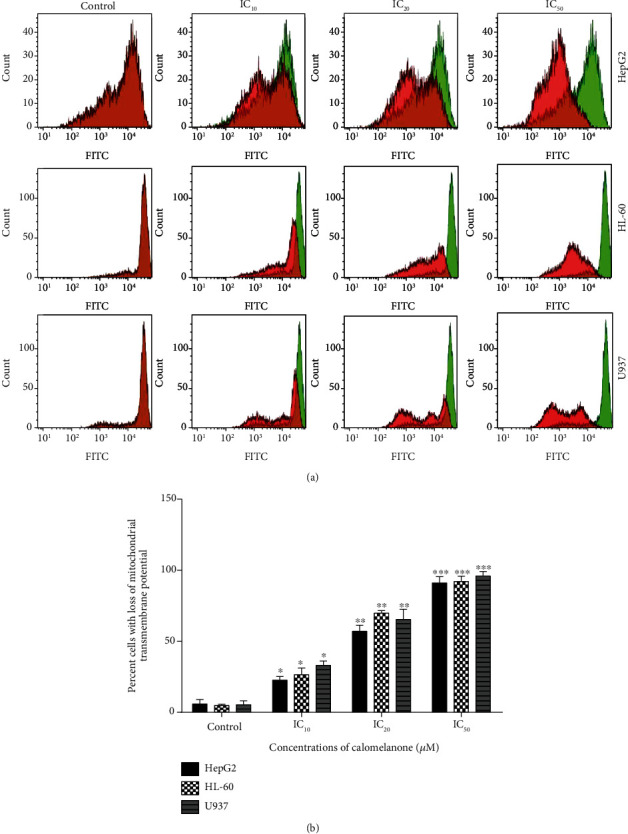
Loss of mitochondrial transmembrane potential (MTP) in calomelanone-treated cancer cells. The leukemic cells were induced by various ICs of calomelanone and treated for 24 h, whereas HepG2 cells were treated with various ICs at 48 h and incubated for 48 h. The cells were then stained with DiOC_6_, and MTP was determined by flow cytometry. Histograms (a) and bar graphs (b) of the percent cells with the loss of MTP are presented as mean values ± SD from three independent experiments. ^∗^*p* < 0.05, ^∗∗^*p* < 0.01, and ^∗∗∗^*p* < 0.001 when compared with the control.

In response to multiple intracellular stress conditions, the channel formation at the outer mitochondrial membrane by Bax/Bak oligomerization is opened, while mitochondrial outer membrane permeabilization (MOMP) is disrupted leading to a decrease in mitochondrial transmembrane potential (MTP) [30]. After treatment, MTP was reduced and the percent cell with a loss of MTP was found by significantly decreasing DiOC_6_ fluorescence intensity. The percentage of cells with the abolishment of MTP increased in a dose-dependent manner in HepG2, HL-60, and U937 cells as shown in Figures 4(a) and 4(b).

### 3.4. Enhancement of Caspase Activities in Calomelanone-Treated HepG2, HL-60, and U937 Cells

**Figure 5:**
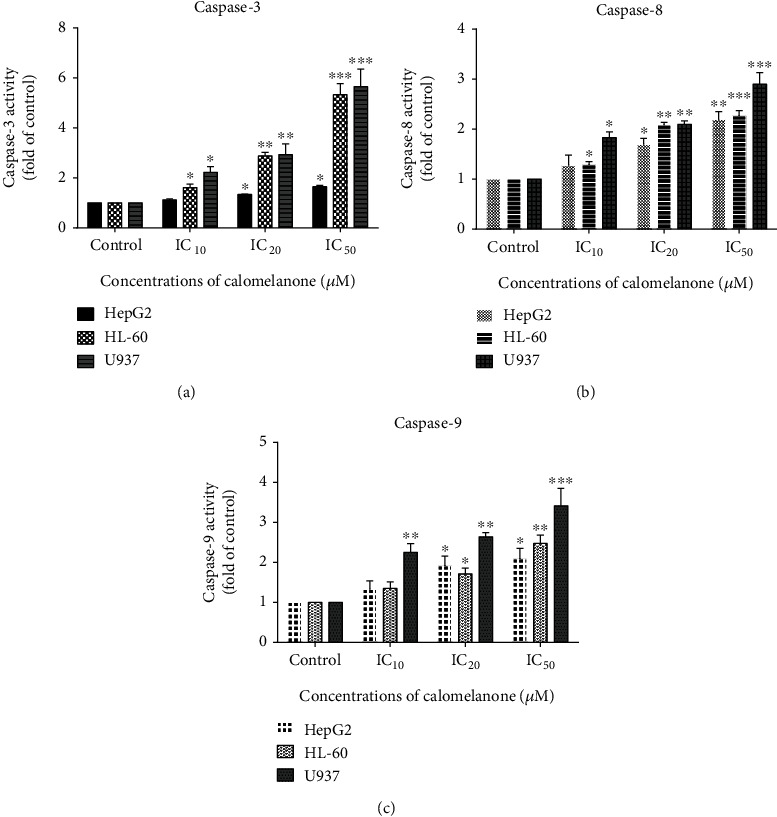
Activation of caspase-3, caspase-8, and caspase-9 activities in calomelanone-treated cancer cells. HL-60 and U937 cells were treated with calomelanone for 24 h, whereas HepG2 cells were treated with calomelanone for 48 h. Caspase-3 (a), caspase-8 (b), and caspase-9 (c) activities were measured by adding each specific substrate that was tagged with *para*-nitroaniline (*p*-NA). The absorbance of the cleaved *p*-NA was measured at 405 nm using a spectrophotometric microplate reader. The caspase activities were then calculated as fold changes and compared to the control. ^∗^*p* < 0.05, ^∗∗^*p* < 0.01, and ^∗∗∗^*p* < 0.001 when compared with the control.

Caspase-8 and caspase-9, initiator proteolytic enzymes, are involved in apoptosis signaling via the death receptor- and mitochondria-mediated pathways, respectively [31]. Calomelanone induced HepG2 cell apoptosis via activation of caspase-8 and caspase-9 (see Figures 5(b) and 5(c), respectively), whereas caspase-3 (Figure 5(a)) was activated in the final common pathway at IC_20_ and IC_50_. The signaling pathways via caspase-3, caspase-8, and caspase-9 were also demonstrated in HL-60 and U937 cells in a dose-dependent manner after treatment with calomelanone (Figures 5(a)–5(c)). It was determined that apoptotic signaling was accomplished through both the intrinsic and extrinsic pathways.

### 3.5. Expression of Bcl-2 Family Proteins by Immunoblotting Analysis

**Figure 6:**
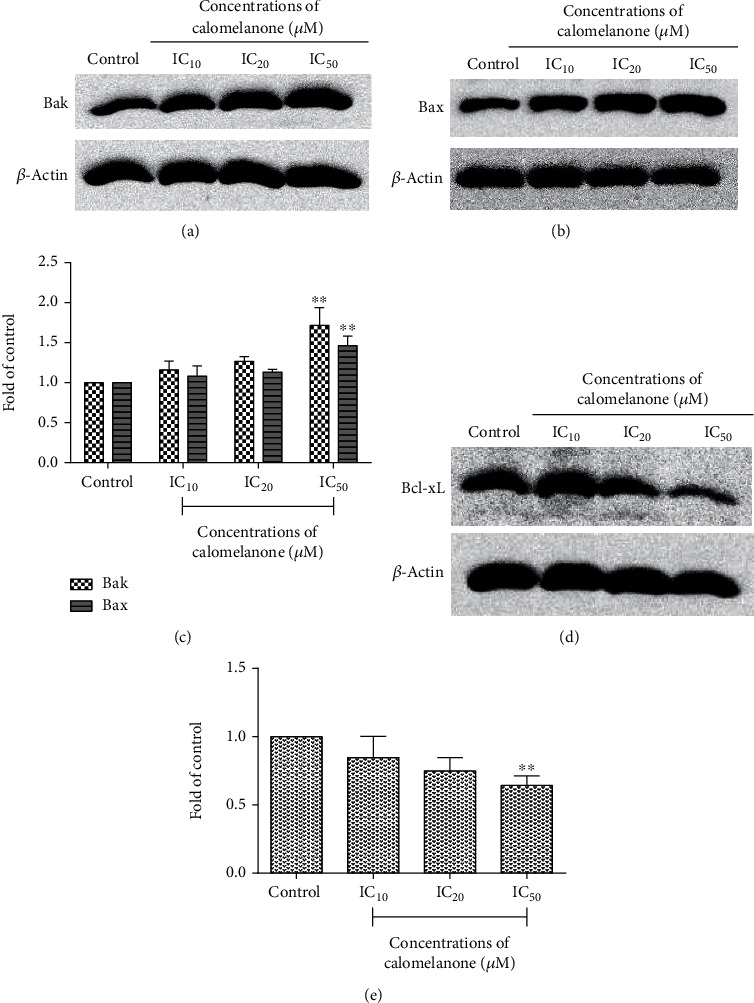
Expression of Bcl-2 family proteins in HepG2 cells after calomelanone treatment. HepG2 cells were treated with calomelanone for 4 h, and the expression levels of Bak and Bax (a–c), as well as those of the antiapoptotic protein Bcl-xL (d, e), were determined by immunoblotting. The protein bands were compared and normalized to *β*-actin as a constitutive protein. Bar graphs represent the protein expression levels that were obtained from three independent experiments with the same results. ^∗∗^*p* < 0.01 when compared with the control.

The Bcl-2 family is involved in the mechanism of apoptotic cell death [32]. Notably, calomelanone could inhibit HepG2 cell proliferation and viability at 48 h but increase cell viability at 24 h. Thus, HepG2 cells were selected for further study. To investigate the molecular mechanism that is relevant to apoptosis, the protein expression levels of the Bcl-2 family were examined. The proapoptotic proteins, including Bak (Figure 6(a)) and Bax (Figure 6(b)), significantly increased at IC_50_ (Figure 6(c)), whereas antiapoptotic protein, Bcl-xL, was found to be significantly reduced after calomelanone treatment at IC_50_ for 4 h when compared to the control (Figures 6(d) and 6(e)).

### 3.6. Production of Reactive Oxygen Species in Calomelanone-Treated HepG2 cells

**Figure 7:**
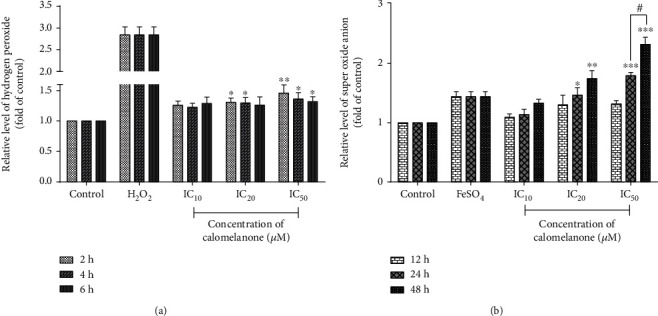
Generation of reactive oxygen species in HepG2 cells by calomelanone. HepG2 cells were treated with calomelanone and stained with DCFH-DA and DHE in order to detect the presence of reactive oxygen species (ROS) including peroxide radicals (a) and superoxide anion radicals (b), respectively. ROS were determined using a fluorescence plate reader. ^∗^*p* < 0.05, ^∗∗^*p* < 0.01, and ^∗∗∗^*p* < 0.001 when compared with the control. ^#^*p* < 0.05 with different times of incubation.

ROS can damage biomolecules, i.e., lipids, proteins, nucleotides (DNA), and organelles, resulting in cellular dysfunction or cell death, as well as apoptosis and autophagy [33]. Calomelanone treatment was observed to cause an increase in hydrogen peroxide or peroxide radicals and superoxide anion radicals, as has been determined by DCFH-DA and DHE, respectively (Figures 7(a) and 7(b)).

### 3.7. Induction of Autophagy in Calomelanone-Treated HepG2 Cells

**Figure 8:**
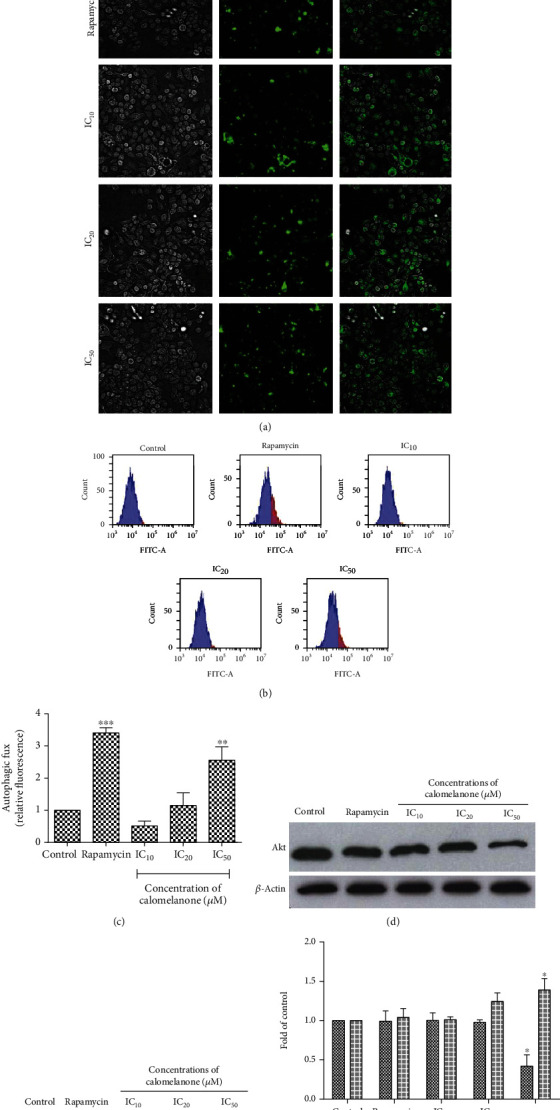
Calomelanone-induced autophagy in HepG2 cells over a short period of incubation. HepG2 cells were treated with calomelanone for 24 h (IC values at 48 h). The degree of fluorescence of the autophagic vacuoles was represented as (1) brightfield, (2) cells stained with monodansylcadaverine (MDC), and (3) cells merged between (1) and (2). The calomelanone-treated HepG2 cells were stained with MDC and examined under a fluorescence microscope (a). The flow cytometry technique was then employed to quantitate the autophagosome/autophagolysosome/autophagic flux. Histograms (b) and bar graphs (c) depict the cells with autophagic flux by increasing fluorescence intensity as mean ± SD values. The survival signaling molecule (Akt) and an autophagic regulatory protein (Atg5) were investigated by Western blotting analysis (d, e), respectively, and the values of mean ± SD are represented as folds of the control (f). Rapamycin was used as a positive control (a–f). ^∗^*p* < 0.05 and ^∗∗^*p* < 0.01 when compared with the control.

Autophagy plays an important role in homeostasis, nutrition, metabolism, and infection-mediated stresses that are known to be initiated by cellular degradation processes [34]. The activation of autophagy depends on the duration of treatment. Autophagy is induced over a short period of treatment time, whereas apoptosis is activated over a longer period of treatment time [35]. Since calomelanone increased HepG2 cell viability at 24 h, the investigation of whether calomelanone induced HepG2 cell survival via the autophagy-mediated mechanism was performed by HepG2 cell treatment at various concentrations of IC values over 48 h but over a shorter incubation period (24 h). At 24 h, the IC values could not be determined due to the proliferation character of HepG2 cells in response to calomelanone. Monodansylcadaverine (MDC) is a specific fluorescence dye for autophagosomes, autophagolysosomes, or autophagic vacuoles, representing the functional autophagic flux [35, 36]. Stained cells, both cell morphology (qualitatively) and quantitatively positive stained cells, were examined under fluorescence microscopy and by flow cytometry, respectively. It was found that autophagy occurred in calomelanone-treated HepG2 cells as shown in (Figure 8(a)). Fluorescence intensity was significantly increased at IC_50_ of the calomelanone treatment when compared with the untreated cells (Figures 8(b) and 8(c)). Atg5, one of the regulators of autophagy [37], was found to be upregulated significantly at IC_50_ (Figure 8(e)). On the other hand, calomelanone reduced the expression of Akt (Figure 8(d)) that is involved in the PI3K/Akt survival pathway.

**Figure 9:**
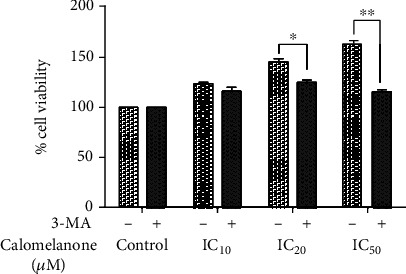
Calomelanone induced autophagy in HepG2 cells for cell survival at 24 h. HepG2 cells were pretreated with the autophagy inhibitor at 5 mM (3-methyladenine (3-MA)) for 1 h. The cells were treated with calomelanone for 24 h (with IC values at 48 h), and the percentage of cell viability was determined by MTT assay. ^∗^*p* < 0.05 and ^∗∗^*p* < 0.01 when compared with the control.

Moreover, HepG2 cells pretreated with 3-methyladenine (3-MA, an autophagy inhibitor) for an hour reduced cell viability significantly. Therefore, 3-MA induced cell death indicating that the autophagy process was a protective pathway for HepG2 cells when treated with calomelanone at 24 h (Figure 9). After 3-MA was withdrawn, autophagy for survival occurred.

## 4. Discussion

There are various natural compounds that possess cytotoxic activity including dihydrochalcones [38]. In 2010, it was reported that calomelanone, 2′,6′-dihydroxy-4,4′-dimethoxydihydrochalcone, could induce prostate cancer LNCaP cell apoptosis via activation of death receptor TRAIL-R [39]. In this study, it was found that calomelanone reduced percent cell viability in human hepatocellular carcinoma HepG2 cells in a dose-dependent manner at 48 and 72 h, whereas it was not found to be toxic against HepG2 cells at 24 h and towards normal human PBMCs at 24 and 48 h. Calomelanone was also found to be toxic to human leukemic HL-60 and U937 cells at 24 h. HL-60 cells were the most sensitive towards calomelanone treatment when compared to U937, HepG2, and PBMC, respectively. This determination was established when IC_50_ and SI values were compared using the same incubation time.

In HepG2 cells, inhibitory concentrations at 10%, 20%, and 50% were selected at 48 h, although the IC values at 72 h were lower because the doubling time of the HepG2 cells was 48 h [40]. It has also been reported that drugs or compound treatments at the doubling time of the cancer cells can improve the sensitivity and cytotoxicity [41, 42]. In addition, limitations of the media can occur when the cell samples are incubated for longer periods of time (72 h). This etiology may make cancer cells vulnerable and die due to a limited surface area and deprivation of nutrients, but not by induction or an effect of the compound [43], which are both considered confounding factors. Moreover, at 72 h of incubation, the media would become acidic due to the presence of the metabolites of the cells, unless new media have been replaced [44]. Thus, IC values at 48 h were selected for further experiments of HepG2 cells, whereas the incubation time for HL-60 and U937 cells was selected at 24 h to investigate the mode of cell death.

Calomelanone significantly induced apoptosis in HepG2, HL-60, and U937 cells. The molecular mechanisms of apoptotic/regulated cell death were then further explored. Calomelanone induced HepG2, HL-60, and U937 cell apoptosis via intrinsic and extrinsic pathways by reducing mitochondrial transmembrane potential (MTP) and inducing caspase-3, caspase-8, and caspase-9 activities. The apoptosis of HepG2 was also confirmed by an increased expression of proapoptotic proteins, i.e., Bax and Bak. On the other hand, expression of the antiapoptotic protein, viz., Bcl-xL, was found to be decreased.

Moreover, a dihydrochalcone analogue, 4′,6′-dihydroxy-2′,4-dimethoxy-5′-(2^″^-hydroxybenzyl)-dihydrochalcone (DHC-2), a purified compound derived from a Thai herb (*Cyathostemma argentatum*), was found to be more sensitive towards HepG2 cells in a dose-dependent manner after incubation for 24 h and at IC_10_, IC_20_, and IC_50_ levels at 23.1 ± 5.3, 45.1 ± 6.3, and 96.2 ± 8.8 *μ*M, respectively (Supplemental Figure 1(b)). DHC-2 also induced HepG2 cells for both early and late apoptosis (Supplemental Figures 2(a) and 2(b)), with the loss of MTP (Supplemental Figures 2(c) and 2(d)) and induction of caspase activities (Supplemental Figures 2(e)–2(g)). Thus, DHC-2, the purified compound obtained from *C*. *argentatum*, induced HepG2 cell apoptosis via both the intrinsic and extrinsic pathways (Supplemental Figure 2). However, DHC-2 has been reported (by our group) to be less toxic towards normal cells, viz., murine fibroblast NIH3T3 cells and normal PBCMs [20].

The generation of reactive oxygen species (ROS) and a lack of antioxidants can cause an imbalance in these two parameters resulting in oxidative stress [45]. ROS are the causative agents that drive the cells to undergo apoptosis/regulated cell death via the intrinsic pathway by inducing the mitochondrial permeability transition pore [46]. It has also been reported that 14 synthesized chalcone derivatives, based on the skeleton of (E)-1,3-diphenyl-2-propene-1-one with three methoxy substituents, can induce HepG2 cell apoptosis via the production of ROS and MTP disruption [47]. Indolyl-chalcone derivatives induce apoptosis by increasing the intracellular ROS level and caspase activities resulting in the suppression of tumor growth in HepG2-xenografted nude mice [48]. In this study, calomelanone significantly induced the production of hydrogen peroxide (peroxide radicals) over a short period of treatment (2-4 h) and superoxide anion radicals over a longer period of treatment (24-48 h). This resulted in the disruption of MTP. ROS can regulate both cell proliferation and cell death depending upon the duration of treatment and the level of ROS. Low levels of superoxide anion radicals can induce cell proliferation, whereas at high levels, cancer cells undergo apoptosis [49]. The level of superoxide anion radicals at 48 h was significantly higher than that at 24 h after calomelanone treatment. Thus, it was confirmed that calomelanone induced HepG2 cell apoptosis/regulated cell death via the mitochondria-mediated pathway and oxidative stress. On the other hand, lower levels of superoxide anion radicals at 24 h of incubation might induce HepG2 cell proliferation/survival instead of cytotoxicity/cell death.

HepG2 cells possess tumor suppressor p53 wild-type proteins, whereas overexpression of MDM2, a p53 inhibitor, occludes p53 activation [50, 51]. Numerous chalcones consist of a number of hydroxy, methoxy, or carboxy groups within their structures that have been reported to influence their level of cytotoxic activity [52]. It has been found that novel chalcones, (E)-1-(2-hydroxy-4-methoxy-3-propylphenyl)-3-(3,4,5-trimethoxyphenyl)prop-2-en-1-one and (E)-3-(3,4-dimethoxyphenyl)-1-(2-hydroxy-4-methoxy-3-propylphenyl)prop-2-en-1-one, which contain various hydroxy and carboxy groups, can induce normal p53 to function by inhibiting p53-MDM2 interaction leading to human non-small-cell lung cancer NCI-H460 cell apoptosis [53]. Chalcones can activate p53 via the interference of p53-MDM2 interaction, which holds two hydrogen bonds between these two peptides/proteins by binding at the p53 binding cleft of MDM2 [54]. Both hydroxy group(s) and methoxy group(s) within the structure of calomelanone (DHC-1) might inhibit p53-MDM2 interaction in the same way as other chalcone derivatives leading to apoptosis/regulated cell death induction. Moreover, O_2_^·−^ significantly increased in calomelanone-treated HepG2 cells and promoted apoptosis via induction of DNA damage and by activating p53 [55]. Therefore, calomelanone might be toxic and induce HepG2 cell apoptosis at 48 h via the same reported binding interaction, which will require further experimentation.

When cells are in the status of stress, such as nutrient deficiency, autophagy plays an important role in degrading the damaged or dysfunctional organelles and aggregated proteins to maintain nutrient recycling, all of which can lead to cellular homeostasis and result in cancer cell survival [34]. Additionally, autophagy can induce the replication of a DNA virus via the upregulation of PI3K class III and/or Beclin-1 in liver cancer, which is also known to cause hepatitis B viral infection [56]. The control of the autophagy mechanism is one of the strategies employed for cancer treatment. Flavokawain B, a chalcone, induces autophagic cell death in gastric cancer cells via induction of ROS, mTOR pathway inhibition, and upregulation of LC3-II accumulation [57]. ROS can activate autophagy through the PI3K/Akt pathway [58]. It has been reported that naringenin, a chalcone, can induce both apoptosis and autophagy in the same cancer cell type via the PI3K/Akt-mediated pathway in human glioblastoma cells [59]. Paratocarpin E, a prenylated chalcone, can induce apoptosis via the intrinsic pathway and activate autophagy by increasing autophagic vacuoles and upregulating autophagic proteins Beclin-1 and LC3-I/II via the p38/ERK/JNK pathway in human breast cancer MCF-7 cells [60]. A novel synthesized chalcone, 3′,5′-diprenylated chalcone, can induce apoptosis via caspase activation and p38/AMPK and ERK1/2 pathways. Moreover, this compound also induces autophagy through the upregulation of LC3-I/II protein expression levels via inhibition of the Akt-mTOR pathway in human leukemic cells [61].

Activation of autophagy depends on the concentration used and the duration of treatment time [35]. Furthermore, a high dose of levofloxacononone, a chalcone derivative, can inhibit autophagy and induce apoptosis of human hepatocellular carcinoma SMMC-7721 cells, whereas the cells are protected by autophagy at low concentrations [62]. Due to the fact that percent cell viability of HepG2 cells was increased after calomelanone treatment at 24 h, we then determined whether HepG2 cells survived via the autophagy mechanism by staining autophagosomes or autophagolysosomes or through measuring autophagic flux by MDC and the expression of autophagic proteins. Calomelanone also induced autophagy in HepG2 cells by suppressing Akt expression, which is known to be an autophagy regulator. Calomelanone also significantly enhanced the expression of the Atg5 protein, which is required for the elongation of the phagophore at IC_50_. Hence, calomelanone upregulated the autophagic protein (Atg5) and suppressed the autophagy-regulated protein, Akt, in HepG2 cells resulting in an increase in the autophagic vacuoles, which is a characteristic feature of autophagic cells.

The autophagy inhibitor, 3-MA, is a viable option for cancer treatment due to its capability of achieving the inhibition of autophagic survival [63]. Notably, it inhibits autophagy via PI3K class III suppression, a survival signaling pathway. A combination of 3-MA with natural product(s) could induce cytotoxicity and apoptosis/regulated cell death in various cancer cell types [64]. In this study, after combining calomelanone with 3-MA, it was found that 3-MA could significantly ameliorate cell viability at high doses (IC_20_ and IC_50_). It has been reported that the combination of a natural compound found in broccoli, sulforaphane, along with 3-MA, could reduce cell viability and induce apoptosis/regulated cell death in human neuroblastoma BE(2)-C cells [65].

Importantly, there is a connection or crosstalk between apoptosis and autophagy pathways. Atg5 can regulate the extrinsic pathway of apoptosis via an interaction with the Fas-associated death domain protein (FADD), which is involved with caspase-8/10 activation [66]. Atg5 is cleaved by calpain to become truncated Atg5 (tAtg5) and is then translocated to mitochondria in order to induce cytochrome c release and drive the cells to undergo apoptosis via the intrinsic pathway [67]. Thus, an increase in the Atg5 expression level by calomelanone treatment might also enhance apoptosis via both the extrinsic and intrinsic pathways.

**Figure 10:**
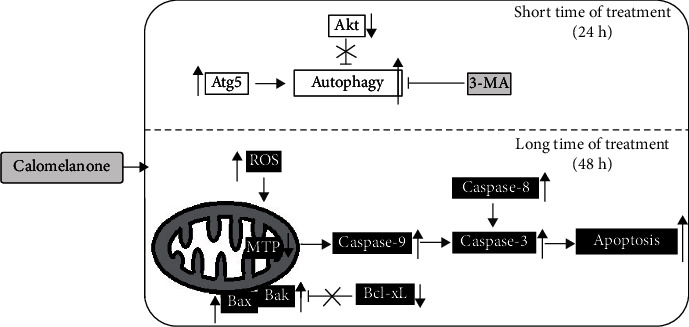
Model representing the mechanisms of calomelanone-induced HepG2 cell apoptosis and autophagy.

Calomelanone (DHC-1) exhibited cytotoxic properties in human hepatocellular carcinoma HepG2, human promyelocytic leukemic HL-60, and human monocytic leukemic U937 cells by inducing apoptosis/regulated cell death through the mitochondrial pathway with a loss of mitochondrial transmembrane potential and an increase in caspase-9 activity. DHC-1 also induced these three cell lines to undergo apoptosis via the extrinsic pathway by the activation of caspase-8. It was found that the Bcl-xL expression level was downregulated, whereas Bax and Bak expression levels were upregulated in calomelanone-treated HepG2 cells. Apoptotic cell death in HepG2 cells was also accomplished via oxidative stress. In addition, autophagy was induced by calomelanone treatment as well as via the inhibition of the Akt pathway and an increase in a specific autophagic protein, Atg5, which resulted in the enhancement of autophagy and survival in HepG2 cells (Figure 10). Importantly, 3-methyladenine could reduce HepG2 cell viability after being treated with calomelanone over a short period of time, which confirmed the survival characteristic of autophagy.

## 5. Conclusion

The present study indicated that calomelanone was capable of inhibiting HepG2, HL-60, and U937 cell proliferation by induction of mitochondria- and death receptor-mediated apoptosis. Calomelanone also induced apoptosis via oxidative stress in HepG2 cells. Moreover, calomelanone at low dose and short duration of treatment, could induce cell survival via autophagy, which 3-MA (an autophagy inhibitor) would inhibit the cell proliferation/survival resulting in human hepatocellular carcinoma HepG2 cell death. Thus, calomelanone possesses the potential to be used as an anticancer agent by itself and/or to enhance the cancer cell death effect in the presence of the autophagy inhibitor, 3-MA.

## Figures and Tables

**Table 1:** Inhibitory concentrations of calomelanone at 10, 20, and 50% in HepG2, HL-60, U937, and PBMCs.

Cancer cells	IC_10_ (*μ*M)	IC_20_ (*μ*M)	IC_50_ (*μ*M)	SI
HepG2 (24 h)	>200	>200	>200	nd
HepG2 (48 h)	23.3 ± 1.3	34.7 ± 1.8^∗^	68.8 ± 4.2^∗∗^	>2.91
HepG2 (72 h)	11.8 ± 2.1	18.4 ± 2.0	39.8 ± 2.9^∗^^,##^	nd
HL-60 (24 h)	28.9 ± 2.1	35.2 ± 1.9	49.4 ± 1.2^∗^^,*δδ*^	>4.05
U937 (24 h)	31.6 ± 2.8	47.1 ± 2.2^∗^	93.3 ± 3.3^∗∗^	>2.14
PBMCs (24 h)	>200	>200	>200	—
PBMCs (48 h)	>200	>200	>200	—

Results are shown as mean ± SD values from triplicate runs of three repeated independent experiments (*n* = 3). ^∗^*p* < 0.05 and ^∗∗^*p* < 0.01 when compared with calomelanone-treated cancer cells at IC_10_ over the same time period and for each individual cancer cell type. ^##^*p* < 0.01 when compared with calomelanone-treated HepG2 cells at IC_50_ treatment for 48 h. *^δδ^p* < 0.01 when compared with calomelanone-treated U937 cells at IC_50_ after 24 h treatment. ^∗^Selectivity index (SI) is the ratio between IC_50_ of PBMCs and IC_50_ of each cancer type at the same duration time of treatment. ^∗^nd = not determined due to IC_50_ values of more than 200 *μ*M and at different time points.

## Data Availability

The data collected in the present study are properly analyzed and summarized in Materials and Methods and Results, and all are available from the corresponding author upon reasonable request. All materials used in this study are properly included in Materials and Methods.

## Supplementary Materials

Supplementary Materials Supplemental Figure 1: cytotoxicity of another dihydrochalcone analogue against the HepG2 cell line. Chemical structure of this dihydrochalcone analogue extracted from a Thai medicinal herb (4′,6′-dihydroxy-2′,4-dimethoxy-5′-(2^″^-hydroxybenzyl)dihydrochalcone, DHC-2) (a). HepG2 cells were incubated with DHC-2 for 24 h, and cell cytotoxicity was measured by MTT assay (b). ^∗∗^*p* < 0.01 and ^∗∗∗^*p* < 0.001. Supplemental Figure 2: the dihydrochalcone analogue (DHC-2)-induced HepG2 cell apoptosis. After HepG2 cells were treated with the dihydrochalcone analogue, 4′,6′-dihydroxy-2′,4-dimethoxy-5′-(2^″^-hydroxybenzyl)dihydrochalcone, at indicated duration times, the mode of cell death was examined by Annexin V-FITC/PI staining. Data are presented in dot plots (a) and bar graphs (b). A loss of mitochondrial transmembrane potential was determined by DiOC_6_ staining, and data are shown in histograms (c) and bar graphs (d). Caspase-3 (e), caspase-8 (f), and caspase-9 (g) activities were measured using each individual specific tetrapeptide substrate tagged with *para*-nitroaniline. The absorbance of the cleaved *p*-NA was then determined using a spectrophotometric microplate reader at 405 nm. ^∗^*p* < 0.05, ^∗∗^*p* < 0.01, and ^∗∗∗^*p* < 0.001.
