# Supplementary material for: Effect of Calomelanone, a Dihydrochalcone Analogue, on Human Cancer Apoptosis/Regulated Cell Death in an In Vitro Model
Source: Biomed Res Int. 2020 Dec 19;2020:4926821. doi: 10.1155/2020/4926821 (PMC7769633; doi:10.1155/2020/4926821)
Supplement: Supplementary Materials — Supplemental Figure 1: cytotoxicity of another dihydrochalcone analogue against the HepG2 cell line. Chemical structure of this dihydrochalcone analogue extracted from a Thai medicinal herb (4′,6′-dihydroxy-2′,4-dimethoxy-5′-(2″-hydroxybenzyl)dihydrochalcone, DHC-2) (a). HepG2 cells were incubated with DHC-2 for 24 h, and cell cytotoxicity was measured by MTT assay (b). ∗∗p < 0.01 and ∗∗∗p < 0.001. Supplemental Figure 2: the dihydrochalcone analogue (DHC-2)-induced HepG2 cell apoptosis. After HepG2 cells were treated with the dihydrochalcone analogue, 4′,6′-dihydroxy-2′,4-dimethoxy-5′-(2″-hydroxybenzyl)dihydrochalcone, at indicated duration times, the mode of cell death was examined by Annexin V-FITC/PI staining. Data are presented in dot plots (a) and bar graphs (b). A loss of mitochondrial transmembrane potential was determined by DiOC6 staining, and data are shown in histograms (c) and bar graphs (d). Caspase-3 (e), caspase-8 (f), and caspase-9 (g) activities were measured using each individual specific tetrapeptide substrate tagged with para-nitroaniline. The absorbance of the cleaved p-NA was then determined using a spectrophotometric microplate reader at 405 nm. ∗p < 0.05, ∗∗p < 0.01, and ∗∗∗p < 0.001. [file 4926821.f1.docx]

## Supplement Data

## Supplementary Figure 1

*Cytotoxic Effect of Another Dihydrochalcone Analogue against Human Hepatocellular Carcinoma (HepG2) Cells.* Another dihydrochalcone analogue, 4',6'-dihydroxy-2',4-dimethoxy-5'-(2''-hydroxybenzyl)-dihydrochalcone (DHC-2), a purified and active compound extracted from a Thai medicinal herb, *Cyathostemma argentatum*, (Supplement Figure 1(a)) reduced percent viability of HepG2 cells in a dose dependent manner after 24 h of treatment (Supplement Figure 1(b)). ICs values (IC_10_, IC_20_, and IC_50_) were 23.1 ± 5.3, 45.1 ± 6.3 and 96.2 ± 8.8 µM, respectively. When compared this dihydrochalcone analogue to calomelanone, this analogue was more toxic against HepG2 cells than calomelanone, concerning the much lower IC_50_ value and sensitivity of less incubation time of this analogue.


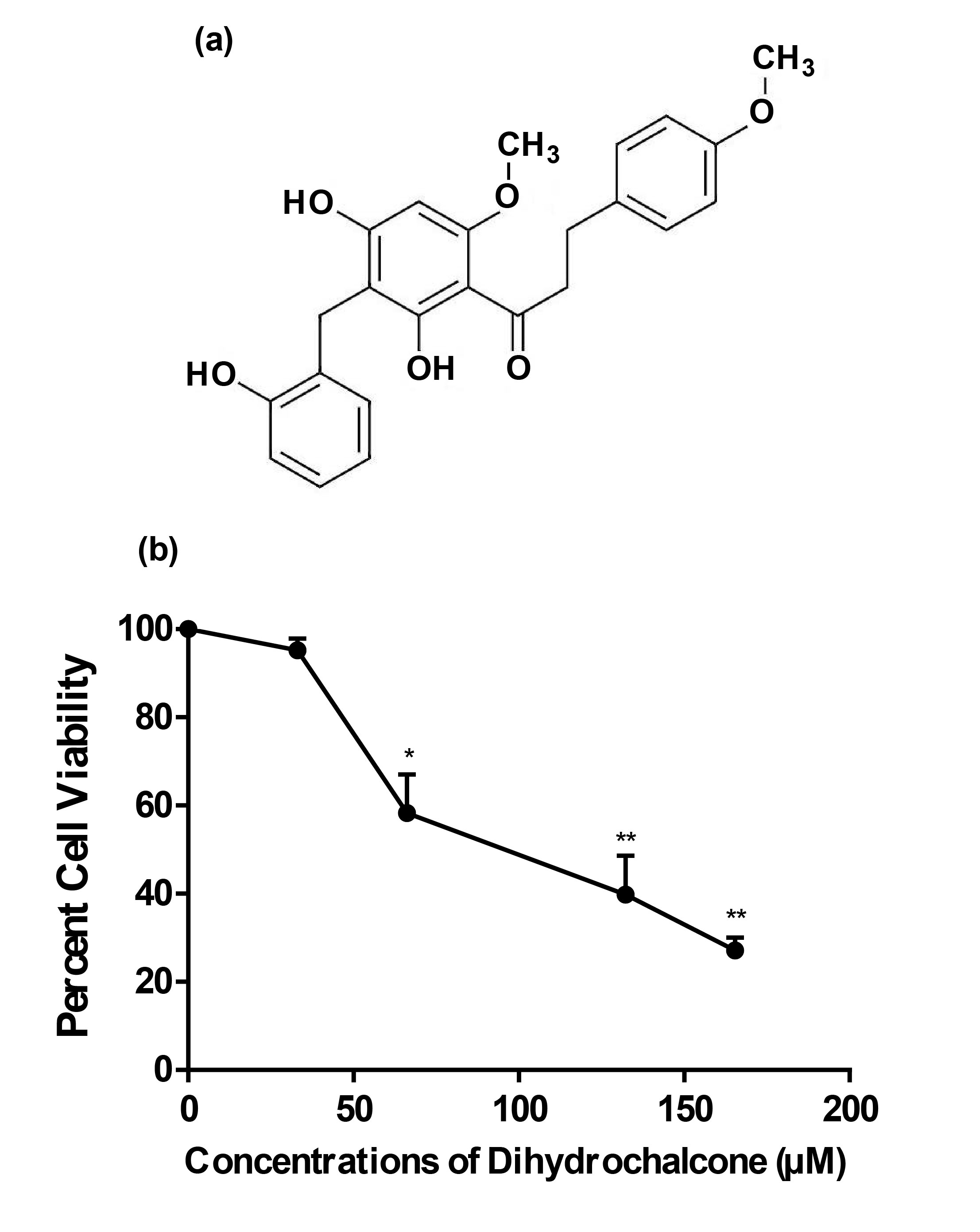


Supplement Figure 1: Cytotoxicity of another dihydrochalcone analogue (DHC-2) against HepG2 cell line. Chemical structure of this dihydrochalcone analogue, extracted from a Thai medicinal herb (a) (4',6'-dihydroxy-2',4-dimethoxy-5'-(2''-hydroxybenzyl)dihydrochalcone). HepG2 cells were incubated with the dihydrochalcone analogue for 24 h, cell cytotoxicity was measured by MTT assay (b). The statistical significance values compared to the control (without treatment) were marked with asterisks, ** *p* < 0.01 and *** *p* < 0.001.

**Supplementary Figure 2**

*Another Dihydrochalcone Analogue-Induced Apoptosis in HepG2 cell line.* After HepG2 cells were treated with the dihydrochalcone analogue, (4',6'-dihydroxy-2',4-dimethoxy-5'-(2''-hydroxybenzyl)dihydrochalcone, DHC-2), at doses of IC_10_, IC_20_ and IC_50_ for 12 and 24 h, the compound induced early apoptosis and late apoptosis in a dose-dependent manner over both duration times (Supplement Figure 2(a) and 2(b)). The compound induced the reduction of the mitochondrial transmembrane potential in both a dose- and time-dependent manner (Supplement Figure 2(c) and 2(d)). In addition, the activities of caspase-3 and -9 were significantly increased at IC_50_ after this dihydrochalcone analogue treatment (Supplement Figure 2(e) and (g), respectively), which confirmed that the analogue induced HepG2 cell apoptosis via the intrinsic pathway. This dihydrochalcone analogue also induced caspase-8 activity at IC_50_ (Supplement Figure 2(f)), which indicated that the dihydrochalcone analogue also induced HepG2 cell apoptosis via the extrinsic pathway.


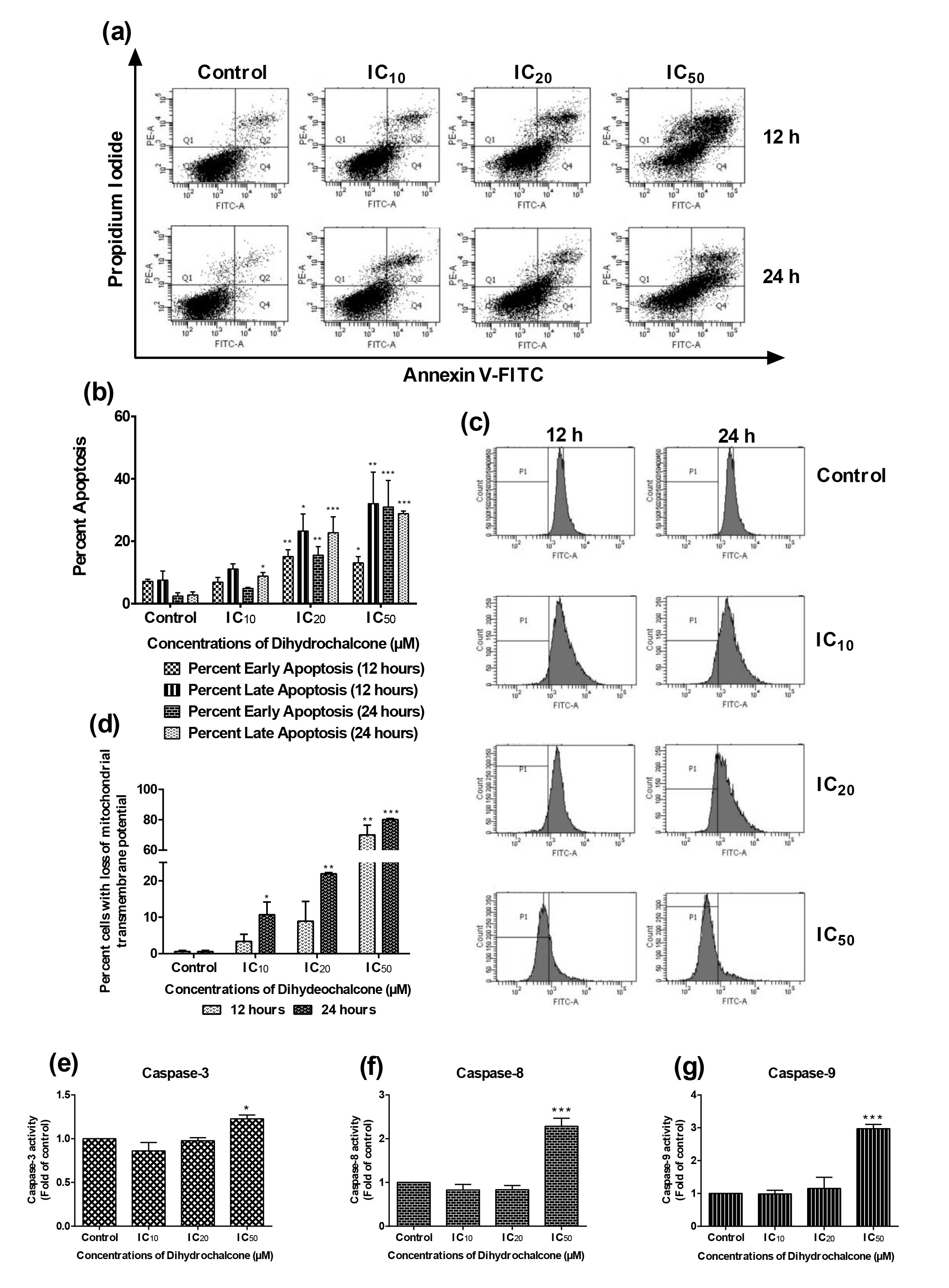


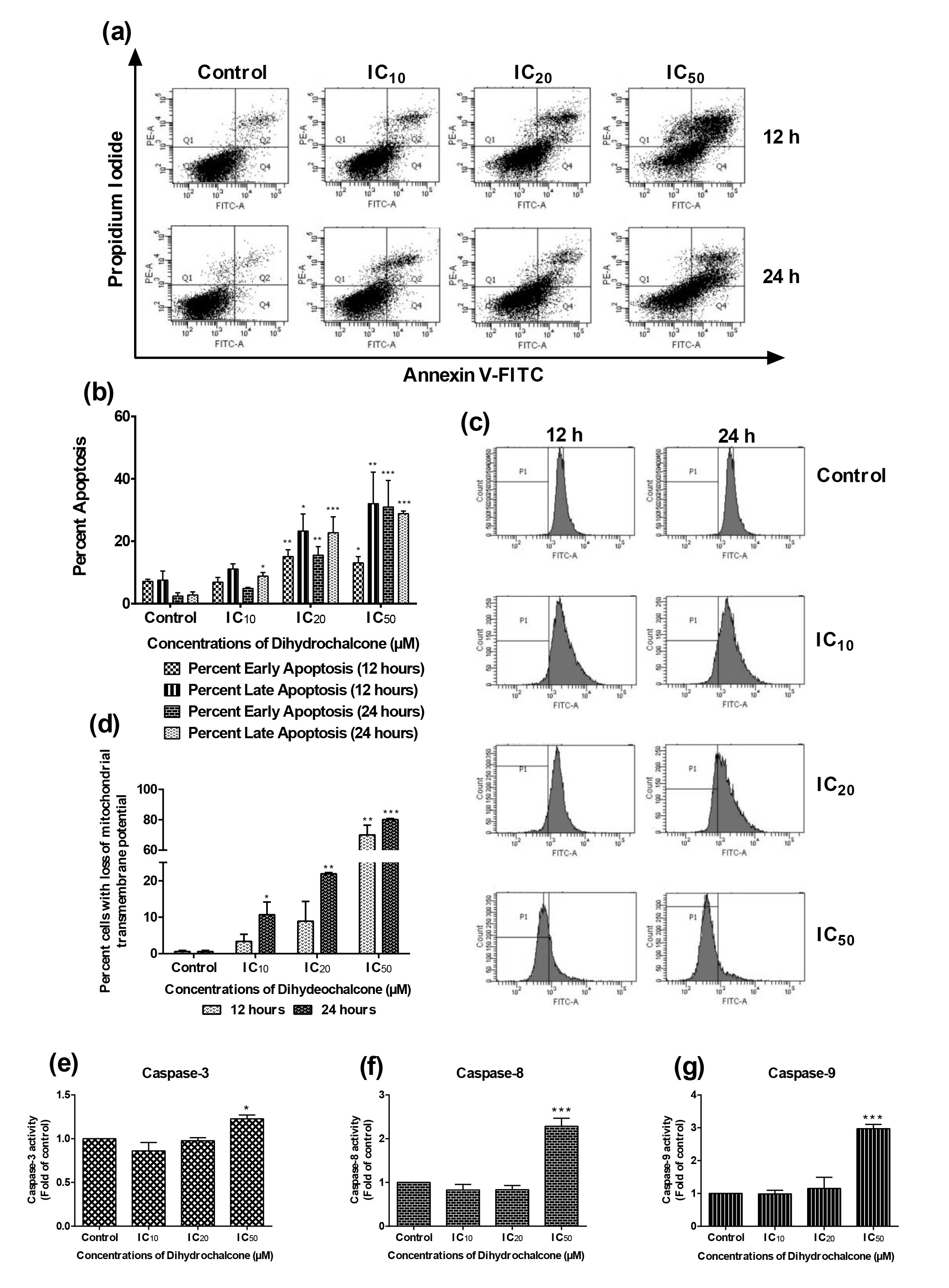


Supplement Figure 2: Another dihydrochalcone analogue-induced HepG2 cells apoptosis. After HepG2 cells were treated with dihydrochalcone analogue, 4',6'-dihydroxy-2',4-dimethoxy-5'-(2''-hydroxybenzyl)dihydrochalcone (DHC-2), at indicated duration times, the mode of cells death was examined by annexin V-FITC/PI staining. Data were represented in dot plots (a) and bar graphs (b). The loss of mitochondrial transmembrane potential was determined by DiOC_6_ staining and data were shown in histograms (c) and bar graphs (d). Caspase-3 (e), -8 (f) and -9 (g) activities were measured using each individual specific tetrapeptide substrate tagged with para-nitroaniline. The absorbance of the cleaved *p*-NA was determined by a spectrophotometric microplate reader at 405 nm. The statistical significance values compared to the control (without treatment) were marked with asterisks, **p* < 0.05, ** *p* < 0.01 and *** *p* < 0.001.
